# The Impact of Apple Variety and the Production Methods on the Antibacterial Activity of Vinegar Samples

**DOI:** 10.3390/molecules26185437

**Published:** 2021-09-07

**Authors:** Mohammed Kara, Amine Assouguem, Omkulthom Mohamed Al kamaly, Safaâ Benmessaoud, Hamada Imtara, Hamza Mechchate, Christophe Hano, Abdou Rachid Zerhouni, Jamila Bahhou

**Affiliations:** 1 Laboratory of Biotechnology, Conservation and Valorisation of Naturals Resources (LBCVNR), Faculty of Sciences Dhar El Mehraz, Sidi Mohamed Ben Abdellah University, BP 1796 Atlas, Fez 30000, Morocco; Safaa.Benmessaoud@usmba.ac.ma (S.B.); zerhounirachid@excite.com (A.R.Z.); jamila.bahhou@gmail.com (J.B.); 2 Laboratory of Functional Ecology and Environment, Faculty of Sciences and Technology, Sidi Mohamed Ben Abdellah University, P.O. Box 2202 Imouzzer Street, Fez 30000, Morocco; Assougam@gmail.com; 3 Department of Pharmaceutical Sciences, College of Pharmacy, Princess Nourah Bint Abdulrahman University, Riyadh 11564, Saudi Arabia; omalkmali@pnu.edu.sa; 4 Faculty of Arts and Sciences, Arab American University Palestine, P.O. Box 240, Jenin 44862, Palestine; 5 Laboratory of Inorganic Chemistry, Department of Chemistry, University of Helsinki, P.O. Box 55, FI-00014 Helsinki, Finland; Hamza.mechchate@helsinki.fi; 6 Laboratoire de Biologie des Ligneux et des Grandes Cultures, INRAE USC1328, University of Orleans, CEDEX 2, 45067 Orléans, France; Christophe.hano@univ-orleans.fr

**Keywords:** antibacterial activity, apple cider vinegar, dietary, the minimum lethal concentration, minimum inhibitory concentration

## Abstract

Apple vinegar is a natural product widely used in food and traditional medicine as it contains many bioactive compounds. The apple variety and production methods are two factors that play a major role in determining the quality of vinegar. Therefore, this study aims to determine the quality of apple vinegar samples from different varieties (Red Delicious, Gala, Golden Delicious, and Starking Delicious) prepared by three methods using small apple pieces, apple juice, and crushed apple, through determining the physicochemical properties and antibacterial activity of these samples. The antibacterial activity was studied against five pathogenic bacteria: *Staphylococcus aureus*, *Klebsiella pneumonia*, *Escherichia coli* (ATB: 57), *Escherichia coli* (ATB: 97), and *Pseudomonas aeruginosa,* using two methods, disk diffusion and microdilution, for determining the minimum inhibitory concentrations and the minimum bactericidal concentrations. The results of this study showed that the lowest pH value was 3.6 for Stark Delicious, obtained by liquid fermentation, and the highest acetic acid values were 4.7 and 4% for the vinegar of Red Delicious and Golden Delicious, prepared by solid fermentation, respectively. The results of the antibacterial activity showed considerable activity of apple vinegar on the tested strains. Generally, the *Staphylococcus aureus* strain appears less sensitive and *Pseudomonas aeruginosa* seems to be very sensitive against all samples, while the other strains have distinct sensitivities depending on the variety studied and the method used. A higher antibacterial activity was found in vinegar obtained by the apple pieces method and the Red Delicious variety, with a low MIC and MBC recorded, at 1.95 and 3.90 µL/mL, respectively. This study has shown that the choice of both apple variety and production method is therefore an essential step in determining and aiming for the desired quality of apple vinegar.

## 1. Introduction

Apple vinegar (AV) is an alcoholic or an acetic fermentation of apple fruits [1], produced either by the traditional method, called the Orleans method, or by the rapid (or submerged) method used in industry using different varieties of apple. Vinegar is considered as a safe and natural food without any additions, as described by GRAS (Generally Recognized As Safe) [2]. It is well-known for its various biological activities, such as antioxidant, antibacterial, and antifungal activity, and its activity on SARS-Cov-1 and SARS-Cov-2 viruses as an effective disinfecting agent to help in preventing the spread and transmission of this new virus, responsible for the COVID-19 pandemic [3], via inhibition, inactivation, and disaggregation of haemagglutinin glycoproteins due to its high acetic acid compound. Additionally, it has low pH-dependent conformational change of those glycoproteins and it destroys the viral envelope and inhibits viral transmission. These properties are the factors influencing the growth of microorganisms, such as bacterial and fungal strains, and as a termiticide [4]. Many studies have shown that it has an effect on diabetes, obesity, cancer, cardiovascular diseases, antitumor properties, and on blood glucose control [5].

Apple vinegar is known for its usefulness as a surface disinfectant of fruits and vegetables or as a natural preservative method for inhibiting the growth of foodborne pathogenic microorganisms in food, and it is used for its multiple antimicrobial potentials with clinical therapeutic implications, such as cleaning and treating nail fungus, head lice, and warts [6,7,8]. Moreover, irrigation of the ear canal with a low concentration of vinegar has positive effects on ear infections, otitis, and myringitis [7,9,10].

Several studies have shown that apple vinegar is very rich in organic acids, phenolic compounds, tannins, flavonoids, and carotenoids, which gives it antioxidant and antibacterial capacity against many pathogenic agents [9,10,11]. Kelebek et al. reported that flavonoids (9.48–62.37 mg/L) and phenolic acids (3.81–21.44 mg/L) were the most abundant compounds contained in the AV [12]. The most important types of flavonoids in AV were flavanols (procyanidin B2, catechins, and epicatechin), flavonols, phloridzin, and phloretin [12,13], and others involving quercetin, camproforol, anthocyanin, cyanidin-3-glucoside, and apicotin [14]. The major phenolic acids found in AV, as reported by several researchers, were gallic acid, chlorogenic acid, caffeic acid, and p-coumaric acids [12,15,16,17]. Indeed, the effect of vinegar on antimicrobial activity is related to its phytochemical compounds [18]. However, the properties of apple vinegar can be due to several factors, such as storage conditions, contact with the wall of the fermentation container, production methods, raw materials, and the length of storage time, which can also contribute to the final quality of vinegars [19]. In Morocco, the vinegar producer cooperatives are based on traditional methods using both liquid and solid-state fermentations. The aim of this study was to characterize and evaluate the impact of the varietal profile and production methods on the antibacterial activity of apple vinegar. In this study, the vinegar is produced by the most popular apple varieties in the region of Imouzzer Kander (Red Delicious, Gala, Golden Delicious, and Starking Delicious) and processed by three methods, using apple pieces (as solid fermentation), apple juice (as liquid fermentation), and crushed apple (as an intermediate state).

## 2. Results and Discussion

### 2.1. Physicochemical Analysis of Samples

The physicochemical properties of our samples are summarized in Table 1. A great diversity was observed in the obtained values. The pH values for all vinegar samples ranged from 3.70 to 5.33. Among the fresh samples, V9 had the highest pH value (5.33) while V12 had the lowest (3.7). The pH values of our samples were very close to each other. The average titratable acidity was calculated as the mass (in grams) of acetic acid equivalent to the total acidity of 100 mL of vinegar in fresh samples at 20 °C. The titratable acidity values ranged from 0.5 to 4.7%, where V1, V7, V10, V12, and V6 samples showed the highest values (4.7, 4, 3.6, 3.1, and 3%, respectively), while the lowest value (0.5%) was indicated in sample V2. Apple cultivar and degree of maturity are among the factors that contribute to the organic acid quantity in apple vinegar, which affects microbiological stability and taste, and therefore acidity and pH. The density of our samples varied between 0.987 and 1.024 g/cm^3^, and the highest value was recorded for V9, while the lowest was recorded for V12. In addition, wide variability in the values of the conductivity of our samples was observed, indicating remarkable differences in vinegar qualities. The highest value was 301 mS/cm (V1) and the lowest was 84 mS/cm (V12). Generally, the conductivity of the vinegar is a result of the presence of suspended mineral matter in the liquid medium. Brix parameter indicates the percentage of soluble solids, including sugar, salts, and proteins, in an aqueous sample. The total soluble solids (TSS) ranged from 1° Brix for V12 and 4.9° Brix for V11. Brix generally represents the sugar equivalents and other soluble materials in the samples that exist in the raw material depending on the cultivar [11,20] Its value is closely related with the fermentation as the level of sugars decreases with the activity of fermentation microorganisms [21]. The alcohol content in our samples of vinegar varied from 0% in V1, V5, and V12 to 1.5% in V11. The value recorded in V11 was higher than that proposed by the Codex Alimentarius Commission, which states that values must be not less than 0.5% [22]. The results showed that the total dry matter (TDM) values of our vinegar samples ranged from 1.52 to 8.51%. The sample V6 showed that the lowest value and the samples V11, V2, and V5 had a high level of TDM (8.51, 2.44, and 2.32%, respectively). From all of these results, it can be concluded that the production methods and the cultivars of apple vinegars showed the important factors that can affect the physicochemical composition properties of vinegars and contribute to the final quality.

### 2.2. Antibacterial Activity

#### 2.2.1. Disc Inhibitory Assay

To evaluate the antibacterial activity of our vinegar samples produced by different apple varieties and with different production methods, we used the disc diffusion test against five strains of studied pathogens. The results obtained by measuring the diameter of the inhibition zone (DIZ) are summarized in Table 2. In general, all strains were sensitive to the vinegar (AV) samples used in our study. The DIZ values for all apple vinegar samples ranged from 6.30 ± 0.58 to 32.70 ± 2.52 mm, and high DIZ values were recorded against *P. aeruginosa* compared to the other strains (*p* < 0.05). V1, V6, and V9 were the samples with the highest antibacterial activity against almost all of the pathogen strains studied. The vinegar prepared by the AP method represents considerable results in the case of the RD variety on all the studied pathogenic germs, whereas the AJ method obtained high positive results for GD against *S. aureus* and *E.coli* (ATB: 57) and *E.coli* (ATB: 97), with a DIZ of 20.70 ± 1.15, 20.70 ± 1.00, and 17.30 ± 0.58 mm, respectively. These results were also observed in the investigation in [7], with DIZ of *S. aureus* and *E. coli* of 28 and 23.5 mm respectively, and according to the results found for traditional vinegar in [23], of 5 and 8 mm, respectively. The obtained results show a significant difference between the GD variety and the varieties RD and Gala. It was reported that acetic acid was the most lethal acid to *Escherichia coli* due to its capacity to pass into cell membranes of microorganisms, leading to bacterial cell death. Most fruit vinegars containing only 0.1% of acetic acid can effectively inhibit the growth of foodborne pathogens in vitro, including *Escherichia coli*, *Staphylococcus aureus* [24], *Pseudomonas aeruginosa*, *Klebsiella pneumoniae,* and others [25]. Generally, the techniques of manufacturing, AP and AJ, can be used for making vinegar from the RD and GD varieties respectively, with high antibacterial activity.

#### 2.2.2. Determination of Minimal Inhibitory Concentration (MIC) and Minimum Bactericidal Concentration (MBC) of Apple Vinegar

According to the results of DIZ, it was observed that apple vinegar affected all microorganisms. The minimal inhibitory concentration (MIC) values of the apple vinegar samples were determined against all bacteria strains. The concentrations used ranged from neat dilution to the lowest of 3.91 μL/mL against each microbe, and the results are shown in Table 3.

The minimum dose required to restrict growth for *S. aureus* was 7.81 µL/mL for V1, V2, V4, V7, and V12; for *K. pneumonia,* it was 3.91 µL/mL for V7 and 7.81 µL/mL for V1, V2, V10, and V12; for *E.coli* 57, it was 7.81 µL/mL for V1, V2, and V4, and 15.62 µL/mL for V7, V10, and V12. For *E. coli* 97, the value of MIC was the lowest, at 1.95 µL/mL for V1 and V2, and *P. aeruginosa* had a MIC of 7.81 µL/mL for V1, V2, V4, V7, and V10, followed by V12 with a MIC of 3.91 µL/mL. In general, all of the strains used were sensitive to our apple vinegar, and a low MIC was clearly observed for V1, V2, V4, V7, V10, and V12. Many studies have shown the effect of AV on the almost pathogenic strains [25]. According to Yagnik et al., the MIC for *C*. *albicans* was required at 1/2 ACV dilution, and for *S. aureus* was 1/25 ACV dilution, whereas for *E. coli* cultures, it was 1/50 ACV dilution [7]. These results can be explained by the high concentration of bioactive compounds existing in most of these cited samples. Acetic acid is one of many organic acids with a high capacity to inhibit the growth of microorganisms at low concentrations [24]. Al-Rousan et al. showed that acetic acid can reduce the growth of *S. Typhimurium* and *E. coli* O157:H7 only at 0.4% [26], and can completely inhibit the growth of *Yersinia enterocolitica* at a concentration of 0.156% (*v/v*) [27]. In addition, several studies have shown that the polyphenols contained in vinegar are also responsible for its higher antibacterial activity. Epicatechin-3-gallate can inhibit 99% of the bacteria strains at a concentration of ≤0.72 mg/mL [28]. Epigallocatechin-3-gallate, reported by Cui et al., can induce the inhibition of *S. aureus* and *S. mutans* at MIC = 0.1 μg/mL, and *E. coli* O157:H7 and *P*. *aeruginosa* at MIC = 0.5 μg/mL [29]. The caffeic acid showed a low effect on various pathogenic strains, with the MIC ranging from 50 to 1024 μg/mL [30,31,32].

The MBC is the bactericidal endpoint that has been subjectively defined as the lowest concentration, at which 99.9% of the final inoculum is killed. The MBC values ranged from 3.91 µL/mL to the pure sample. The lowest values were 3.91 µL/mL for V1 against *E. coli* (ATB: 97) and 7.81 µL/mL for V1 and V2 against *P. aeruginosa* and *E.coli* (ATB: 97). In general, it was clearly observed that V1 and V2 are the samples which can kill all of the strains studied at doses lower than 15.62 µL/mL, whereas the other samples had a bactericidal effect at MBC higher than 15.62 µL/mL. Moreover, V5 and V6 are considered as the sample with lower antibacterial activity.

It has been shown above that each sample of apple vinegar has an antibacterial effect on several types of bacteria. The antibacterial effect is mainly due to acetic acid and polyphenolic compounds [9], which can stop the growth of bacteria strains or lead to their death by modifying the condition of the medium and the essential mechanisms responsible for their growth, and acting as an anti-germination agent [4]. Cushnie et al. reported that flavonoids preventing the development of antibiotic resistance in bacteria have an ability to inactivate the efflux pump, destabilize the cytoplasmic membrane, and inhibit β-lactamases and topoisomerase enzymes secreted by bacteria [33]. Catechins induce damage on lipopolysaccharide, acting as a barrier in the bacterial membranes [34].

The decrease in antibacterial activity may be due to the decrease in bioactive compounds in the final vinegar products. In this study, the filtration of apples during the first stage of vinegar production led to the loss of many bioactive molecules. Francini and Sebastiani found that polyphenols can reside in apple pomace after juice extraction [35]. Procyanidins and tannins reside in parietal surfaces in association with polysaccharides and protein structures by non-covalent bonds [36,37]. This allows us to conclude that the method based on the apple pieces (AP) and the one using crushed apple (CA) can keep a maximum of bioactive compounds in vinegar that are responsible for its microbiological activities. In Morocco, the production of commercial vinegar is performed by the fast method using apple juice. On the other hand, the traditional method is often limited to the production of home-made vinegar or used by vinegar producer cooperatives. Based on these results, it can be proposed that the use of crushed apple can take place instead of apple juice as a method to avoid the loss of bioactive compounds and consequently to ensure a better antibacterial activity. In addition, the Red Delicious variety can be used to produce apple vinegar owing to its antibacterial activity and physicochemical proprieties.

### 2.3. Correlation between Investigated Quality Parameters of the Vinegars

**Figure 1:**
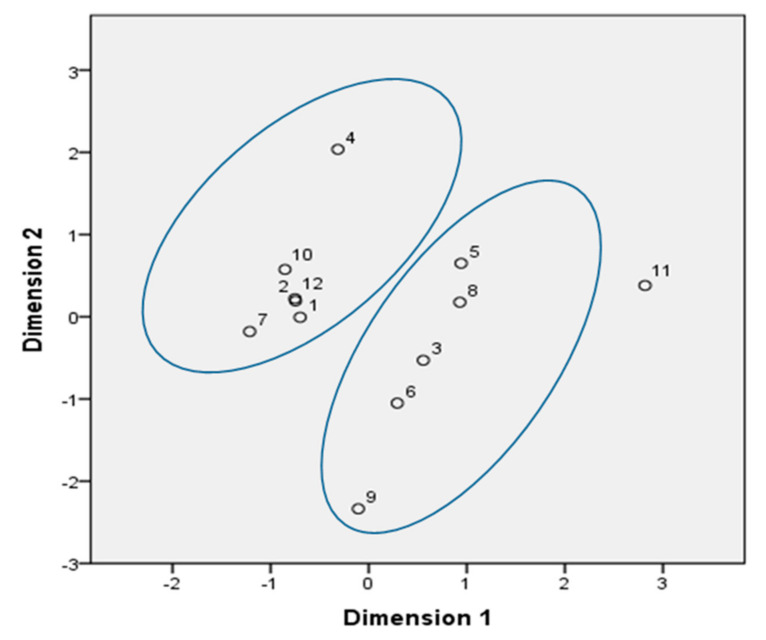
Multiple correspondence analysis of all samples based on the function of their studied properties.

The correlation between investigated quality parameters of all the vinegar samples was calculated by Pearson’s correlation and presented in Table 4. Statistically significant correlations were established between chemical quality characteristics, with the highest positive correlation between TSS and content of alcohol (r = 0.99), and between density and alcoholic content, TSS, pH, and conductivity (r = 0.61, r = 0.68, r = 0.74, and r = 0.71, respectively). The content of dry matter negatively correlated with vinegar antimicrobial activity to *S. aureus* (r = −0.83), while acetic acid correlated positively (r = 0.61) with *K. pneumonia*. The latter correlation is reasonable, because acetic acids which possess antimicrobial potential correlated with the pH value. Thus, apple vinegar is a food rich in phenolic compounds. The kind and quantity of each phenolic substance present in vinegar manifested different levels of respective activity [24]. A correlation was also found between *E. coli* (ATB: 57) and *E. coli* (ATB: 97), with r = 0.84, probably due to their similar membrane characteristics, while *K. pneumonia* was positively correlated with *P. aeruginosa* (r = 0.77). The samples used in this study can be labeled in two major categories based on the function of their properties studied (Figure 1).

## 3. Materials and Methods

### 3.1. Preparation of Samples

**Figure 2:**
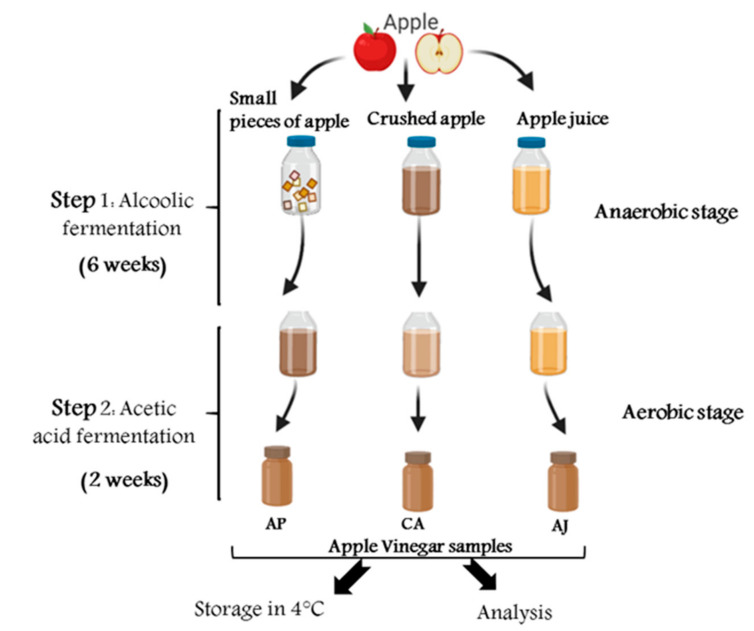
Summary of apple vinegar processing methods used in this study.

Four of the most commonly planted apple varieties in Morocco were selected: Red Delicious (RD), Gala (Gala), Golden Delicious (GD), and Starking Delicious (SD). The four varieties were harvested in early September from the same apple yard located in the rural commune of Ait Sebaa, in the region of Sefrou (33°73′09.36′′ N; 5°02′35.26′′ W), Morocco. Each of the four varieties of apples were lightly washed and dried before fermentation. The apple vinegar was made using three different methods: the first consists of using the apples in the form of small pieces (AP), the second is based on apple juice (AJ), and the third uses crushed apple (CA). The alcoholic fermentation was carried out in hermetically sealed bottles for 40 days at room temperature (20 ± 3 °C). The small pieces of apples used in the AP method were filtered under pressure in the first stage, during the acetic fermentation, while the fermented mash was filtered for alcoholic fermentation in aerobic conditions over 2 weeks. Fresh samples obtained from this fermentation were frozen at 4 °C for later analysis (Figure 2).

### 3.2. Physiochemical Analysis

The pH was measured by direct reading using a previously calibrated pH Bench Meter pH 210 for Quality Control Applications (Hanna Instruments, Washington, DC, USA). The titratable acidity of vinegar was calculated as the percentage (%) of acetic acid [38]. A handheld refractometer (0–40% Brix) (Model ATAGO POCKET REFRACTOMETER) (ATOGO, Tokyo, Japan) was used to measure the soluble solid content (TSS) (°B) and residual alcohol (%) of vinegar [39]. The conductivity was measured by a conductivity meter and expressed in mS/cm. The density (g/cm^3^) was determined using density meter instruments. Total dry matter (%) was carried out according to Association of Official Analytical Chemists methods [40].

### 3.3. Quantitative Assessment of Antibacterial Activity of Apple Vinegar

In this study, the antibacterial activity of apple vinegar was tested against five pathogenic bacteria responsible for several infections. Both *Escherichia coli* (ATB: 57) B6N (Gram−) and *Escherichia coli* (ATB: 97) BGM (Gram−) were obtained from the Hassan II University Hospital of Fez, and *Pseudomonas aeruginosa* (Gram−), *Klebsiella pneumonia* (Gram−), and *Staphylococcus aureus* (Gram+) from the laboratory of microbiology, Faculty of Medicine and Pharmacy, Fez, and all were used as test microorganisms. The cultures were stored on Mueller–Hinton (MH) agar in the refrigerator (4 °C).

#### 3.3.1. Bacterial Strains and Inoculums Standardization

The different bacterial strains were transplanted by the streak method into petri dish MH agar and incubated in an oven at 37 °C for 18 to 24 h in order to obtain a young culture and isolated colonies. An isolated colony of each strain was collected using a platinum loop and homogenized in sterile saline (0.9% NaCl), and incubated for 3–5 h at 37 °C for pre-culture, then it was adjusted to the turbidity 0.5 McFarland standard (equivalent to 1–5 × 10^8^ CFU/mL) [41].

#### 3.3.2. Agar Well Diffusion (AWD) Assay

The agar well diffusion assay is based on the method of Kirby-Bauer [42], with slight modifications. An autoclaved cotton swab was dipped into the standardized suspension (1–5 × 10^8^ CFU/mL) and rubbed horizontally across the surface of the labeled Mueller–Hinton agar (MHA) plates to conduct lawn culture of the bacterial strains. Whatman paper discs (6 mm) were placed on the surface of pre-inoculated agar and impregnated with 10 µL of AV. After incubation of all plates at 37 °C for 24 h, the diameters of the inhibition zones were determined. All the tests were conducted 3 times to obtain the mean value of zones of inhibition, which was then used to calculate the standard deviation value.

#### 3.3.3. Determination of the Minimum Inhibitory Concentration (MIC)

The MICs were determined by microdilution assays in 96-well plates to determine the minimum concentration of vinegar needed to inhibit the pathogen strains used in our study according to the standards of the NCCLS [43], with slight modifications. Ten concentrations of AV were disposed in sterile tubes, carried out by successive 1/2 dilutions in distilled water. The concentrations of AV obtained in the well were between 0.975 and 500 µL/mL. Preparation of bacterial suspensions was performed as described previously [41]. Briefly, 20 µL of these suspensions were diluted in 80 µL of liquid MH broth and placed in 96-well plates at a density of 5.0 × 10^5^ CFU/well. Finally, 100 µL of different concentrations of AV were added to each well to determine the MIC values. After 24 h of incubation at 37 °C, the colorimetric method based on the use of dye reagents (Triphenyltetrazolium chloride (TTC)) was used. After 2 h of incubation, the MIC was determined as the lowest concentration that does not produce red color with a high ability to detect strain growth in the wells [44].

#### 3.3.4. Determination of the Minimum Bactericidal Concentration (MBC)

The determination of the minimum bactericidal concentration (MBC), also called the minimum lethal concentration (MLC), was performed as described in document M26-A [45], with minor modifications. Briefly, using a cotton swab, three wells were taken, juxtaposed with those of the MIC. After their growth on the surface of the non-selective agar dish, the number of surviving cells (CFU/mL) after 24 h of incubation must be determined. The bactericidal endpoint (MBC) was subjectively defined as the lowest concentration at which 99.9% of the final inoculum is killed [46].

### 3.4. Statistical Analysis

Data were expressed as mean and standard deviation (SD). A one-way analysis of variance was used to analyze data, with *p* < 0.05 representing a significant difference between means, as estimated with a multiple range test using the least significant difference (LSD) or Duncan’s test at α < 0.05. Multiple correspondence analysis was used to determine the homogeneous subgroups.

## 4. Conclusions

The results of this study show that all the samples have inhibitory activity against all the bacterial strains tested at different concentrations. The results showed that the manufacturing methods can influence the quality of vinegar and can affect the physicochemical composition properties of vinegar and contribute to its antibacterial activity. The lowest pH value was 3.7, for vinegar based on the juice of the Starking Delicious variety. The Red Delicious (RD), Gala (Gala), and Golden Delicious (GD) prepared by apple pieces, and Golden Delicious (GD) and Starking Delicious (SD) fermented in a liquid state as juice, showed the highest values of acetic acid, while the vinegar prepared by pieces of the Red Delicious variety had a stronger antibacterial activity than the other samples. In general, the V1, V2, V4, V7, V10, and V12 samples had similar properties and can be used to produce a vinegar with high physiochemical characteristics and antibacterial activity. The combination of apple variety and production technique is therefore an essential step for determining the final quality of apple vinegar, which can be used in medicinal plant therapy. Therefore, future studies are needed to fully understand the vinegar production process and how it influences its compounds.

## Figures and Tables

**Table 1:** Physicochemical characteristics of different samples of vinegar prepared by different methods and apple varieties.

Varieties	Manufacturing Techniques	Code of Simple	Acetic Acid (%)	Density (g/cm^3^)	Alcohol Content (%)	Total Dry Matter (%)	TSS (°B)	pH	Conductivity (mS/cm)
RD	AP	V1	4.7	1.003	0	2.45	1.5	4.6	301
	CA	V2	0.5	1.005	1	3.44	4	4.96	287
	AJ	V3	0.7	1.011	0.5	ND	1.2	5.02	298
Gala	AP	V4	0.5	1.015	1.3	2.68	4.2	5.06	284
	CA	V5	0.6	1.010	0	3.32	1.5	5.29	266
	AJ	V6	3.0	1.013	1	1.52	4	4.84	296
GD	AP	V7	4.0	1.023	1	2.22	4	5.11	281
	CA	V8	0.9	1.006	1	3.4	4	5.08	289
	AJ	V9	1.0	1.024	1	2.59	4	5.33	266
SD	AP	V10	3.6	1.015	0.7	3.30	3	5.15	278
	CA	V11	1.0	1.012	1.5	8.51	4.9	4.02	260
	AJ	V12	3.1	0.987	0	2.07	1	3.7	84

RD: Red Delicious; GD: Golden Delicious; SD: Starking Delicious; AP: apple pieces; CA: crushed apple; AJ: apple juice. ND: not detected.

**Table 2:** Diameters of inhibition zones (mm) of apple vinegar relative to the bacteria tested.

Varieties	Manufacturing Techniques	Code of Sample	Diameter of Inhibition Zone (mm) #				
			*S. aureus*	*K. pneumonia*	*E.coli* (ATB: 57)	*E.coli* (ATB: 97)	*P. aeruginosa*
RD	AP	V1	19.00 ^ab^ ± 1.00	17.30 ^a^ ± 0.58	15.00 ^b^ ± 1.00	14.00 ^ab^ ± 1.00	32.70 ^a^ ± 2.52
	CA	V2	15.70 ^bc^ ± 1.10	14.70^ab^ ± 0.58	11.70 ^bc^ ± 1.53	13.70 ^bc^ ± 1.15	25.70 ^bc^ ± 1.15
	AJ	V3	12.00 ^f^ ± 0.00	13.30 ^d^ ± 0.58	10.70 ^c^ ± 1.15	9.00 ^e^ ± 1.00	10.70 ^d^ ± 1.15
Gala	AP	V4	11.30 ^d^ ± 1.15	11.30 ^c^ ± 1.15	12.00 ^bc^ ± 0.00	10.70 ^cde^ ±1.15	30.00 ^ab^ ± 0.00
	CA	V5	10.70 ^d^ ± 0.58	11.00 ^c^ ± 1.00	10.30 ^c^ ± 0.58	10.00 ^de^ ± 0.00	13.00 ^d^ ± 0.00
	AJ	V6	15.70 ^bc^ ± 1.15	15.70 ^a^ ± 2.52	15.30 ^b^ ± 2.52	14.00 ^ab^ ± 1.73	22.00 ^c^ ± 1.73
GD	AP	V7	14.00 ^cd^ ± 1.73	12.30 ^bc^ ± 2.08	11.30 ^bc^ ± 3.05	13.00 ^bcd^ ±1.00	21.00 ^c^ ± 3.61
	CA	V8	12.00^cd^ ± 0.00	10.00 ^c^ ± 0.00	12.30 ^bc^ ± 0.58	12.00 ^cde^ ± 1.73	10.00 ^d^ ± 0.00
	AJ	V9	20.70 ^a^ ± 1.15	6.30 ^d^ ± 0.58	20.70^a^ ± 1.00	17.30 ^a^ ± 0.58	12.70 ^d^ ± 1.15
SD	AP	V10	11.30^d^ ± 1.15	14.70^ab^ ± 1.53	10.00 ^c^ ± 0.00	12.70 ^cd^ ± 1.53	21.30 ^c^ ± 1.15
	CA	V11	13.00^g^ ± 0.00	10.00 ^c^ ± 0.00	15.30 ^b^ ± 1.15	16.30 ^ab^ ± 1.15	12.70 ^d^ ± 0.58
	AJ	V12	13.30^cd^ ± 2.89	12.30 ^bc^ ± 0.58	10.00 ^c^ ± 0.00	10.00 ^de^ ± 0.00	20.00 ^c^ ± 5.00

a, b, c, d, e, f, and g denote that a significant difference exists between means of antibacterial test results of apple vinegar samples (*p* < 0.05) according to Tukey’s LSD test, where “a” denotes the highest mean value and “g” the lowest mean value. #: mean ± SD. RD: Red Delicious; GD: Golden Delicious; SD: Starking Delicious; AP: apple pieces; CA: crushed apples; AJ: apple juice.

**Table 3:** Minimal inhibitory concentration (MIC) and minimum bactericidal concentration (MBC) of apple vinegar relative to the bacteria tested.

Varieties	Manufacturing Techniques	Code of Sample	MIC and MBC (µL/mL)									
			*S. aureus*		*K. pneumonia*		*E.coli (ATB: 57)*		*E.coli (ATB: 97)*		*P. aeruginosa*	
			MIC	MBC	MIC	MBC	MIC	MBC	MIC	MBC	MIC	MBC
RD	AP	V1	7.81	15.62	7.81	15.62	7.81	15.62	1.95	3.91	7.81	7.81
	CA	V2	7.81	15.62	7.81	15.62	7.81	15.62	1.95	7.81	7.81	15.62
	AJ	V3	125	62.5	125	31.25	62.5	31.25	31.25	31.25	31.25	31.25
Gala	AP	V4	7.81	31.25	15.62	31.25	7.81	31.25	7.81	15.62	7.81	15.62
	CA	V5	62.5	125	500	500	500	500	500	500	500	500
	AJ	V6	500	500	250	250	500	500	500	500	250	250
GD	AP	V7	7.81	31.25	3.91	15.62	15.62	31.25	7.81	31.25	7.81	31.25
	CA	V8	62.5	62.5	62.5	31.25	62.5	31.25	31.25	31.25	31.25	31.25
	AJ	V9	31.25	62.5	31.25	62.5	31.25	125	62.5	125	31.25	125
SD	AP	V10	15.62	62.5	7.81	31.25	15.62	31.25	7.81	31.25	7.81	31.25
	CA	V11	ND	ND	500	ND	500	ND	500	ND	500	ND
	AJ	V12	7.81	31.25	7.81	31.25	15.62	62.5	15.62	31.25	3.91	15.62

RD: Red Delicious; GD: Golden Delicious; SD: Starking Delicious; AP: apple pieces; CA: crushed apples; AJ: apple juice; ND: not detected.

**Table 4:** Pearson correlation coefficients between different parameters of apple vinegar.

	Acetic Acid	Density	Alcoholic Strength in %	Total Dry Matter	TSS (°B)	pH	Conductivity (mS/cm)	*S. aureus*	*K. pneumonia*	*E. coli* (ATB: 57)	*E. coli* (ATB: 97)	*P. aeruginosa*
Acetic acid	1.0000											
Density	−0.1646	1.0000										
Alcoholic strength in %	−0.4109	0.6104	1.0000									
Total dry matter	−0.3886	0.0654	0.4328	1.0000								
TSS (°B)	−0.3891	0.6780	0.9866	0.3963	1.0000							
pH	−0.2069	0.7404	0.1485	−0.3567	0.2390	1.0000						
Conductivity (mS/cm)	−0.0759	0.7064	0.4134	0.0718	0.5086	0.6939	1.0000					
*S. aureus*	0.2993	0.0394	−0.3536	−0.8296	−0.2729	0.4049	0.1045	1.0000				
*K. pneumonia*	0.6094	−0.3944	−0.3292	−0.3032	−0.3036	−0.1257	0.1997	0.3788	1.0000			
*E. coli* (ATB: 57)	−0.1348	0.4218	0.3424	0.1144	0.4001	0.1123	0.2964	0.3657	−0.1771	1.0000		
*E. coli* (ATB: 97)	−0.0438	0.4793	0.5191	0.3875	0.5895	0.0605	0.4013	0.2628	0.0996	0.8360	1.0000	
*P. aeruginosa*	0.3770	−0.2465	−0.1129	−0.4278	−0.1309	0.0048	0.2349	0.4518	0.7677	−0.0769	0.0208	1.0000

## Data Availability

The data presented in this study are available upon request.

## Sample Availability

Samples are available from the authors upon reasonable request.
